# Cloning and expression of the sucrose phosphorylase gene in *Bacillus subtilis* and synthesis of kojibiose using the recombinant enzyme

**DOI:** 10.1186/s12934-017-0842-2

**Published:** 2018-02-15

**Authors:** Miaomiao Wang, Jing Wu, Dan Wu

**Affiliations:** 1 State Key Laboratory of Food Science and Technology, Jiangnan University, 1800 Lihu Avenue, Wuxi, 214122 China; 2 School of Biotechnology and Key Laboratory of Industrial Biotechnology, Ministry of Education, Jiangnan University, 1800 Lihu Avenue, Wuxi, 214122 China; 3 International Joint Laboratory on Food Safety, Jiangnan University, 1800 Lihu Avenue, Wuxi, 214122 China

**Keywords:** Kojibiose, Sucrose phosphorylase, *Bacillus subtilis*, Enzymatic transformation, Heterologous expression

## Abstract

**Background:**

Kojibiose as a prebiotic and inhibitor of α-glucosidase exhibits potential for a wide range of applications in the food and medicine fields; however, large-scale separation and extraction of kojibiose from nature is difficult. Sucrose phosphorylase (SPase) can be used for the production of kojibiose, and currently, SPase is only heterologously expressed in *E. coli*, making it unsuitable for use in the food industry. However, *Bacillus subtilis* is generally considered to be a safe organism potentially useful for SPase expression.

**Results:**

Here, for the first time, we heterologously expressed *Bifidobacterium adolescentis* SPase in a food-grade *B. subtilis* strain. The results showed that SPase was efficiently secreted into the extracellular medium in the absence of a signal peptide. After culturing the recombinant strain in a 3-L bioreactor, crude SPase yield and activity reached 7.5 g/L and 5.3 U/mL, respectively, the highest levels reported to date. The optimal reaction conditions for kojibiose synthesis catalyzed by recombinant SPase were as follows: 0.5 M sucrose, 0.5 M glucose, 0.02 U_enzyme_/mg_all_substrates_, pH 7.0, 50 °C, and 30 h. Furthermore, the substrate-conversion rate reached 40.01%, with kojibiose accounting for 104.45 g/L and selectivity for kojibiose production at 97%.

**Conclusions:**

Here, we successfully expressed SPase in *B. subtilis* in the absence of a signal peptide and demonstrated its secretion into the extracellular medium. Our results indicated high levels of recombinant enzyme expression, with a substrate-conversion rate of 40.01%. These results provide a basis for large-scale preparation of kojibiose by the recombinant SPase.

## Background

Kojibiose (2-O-α-d-glucopyranosyl-α-d-glucopyranose) is a natural disaccharide comprising two glucose moieties linked by an α-1,2 glycosidic bond. Kojibiose and kojibiose-derived oligosaccharides are not easily digested and can prevent tooth decay [1]. Because the human intestinal system tolerates kojibiose, and kojibiose is a proliferation factor for *Bifidobacterium,* lactic acid bacteria, and eubacteria, it represents a good prebiotic element. Additionally, kojibiose is a low-calorie sweetener capable of increasing the absorption of iron [2]. Furthermore, kojibiose can specifically inhibit the activity of α-glucosidase I in different tissues and organs [3–5] and exhibits antitoxic activity, thereby promoting the emergence of new drugs, such as anti-HIV-I drugs, and leading to an increased interest in kojibiose.

Methods for kojibiose preparation mainly include chemical extraction and enzymatic synthesis. In nature, kojibiose mainly exists in beer, honey, sake, koji extract, fecal streptococci, molasses, and starch hydrolysates. Herbs, such as *Pseudostellariae* and *Aralia elata*, also contain kojibiose; however, the content of natural kojibiose is very low, resulting in difficult separation and purification [6–8] and making large-scale extraction of kojibiose problematic. Enzymes necessary for kojibiose synthesis mainly include sucrose phosphorylase (SPase; EC 2.4.1.7) [9], β-galactosidase (β-gal), dextranase [10], kojibiose phosphorylase [11], and α-glucosidase [12, 13]. SPase uses inexpensive sucrose and glucose as substrates for kojibiose synthesis, making it an attractive option for market development.

SPase belongs to the GH13 family and is capable of catalyzing transglycosylation [14]. SPase mainly catalyzes two types of reactions: one transfers the glucosyl moiety of 1-phosphate glucose to an acceptor, and the second transfers the glucosyl moiety of sucrose to an acceptor. The acceptors consist of inorganic phosphoric acid, water, and substances containing phenolic hydroxyl, alcoholic hydroxyl, or carboxyl groups [15]. When glucose is used as an acceptor, maltose and kojibiose are generated. SPase from *Bifidobacterium adolescentis* exhibits high transglycosylation activity and utilizes a wide range of acceptors when sucrose is used as the glycosyl donor [16]. In 2004, Sprogøe et al. [17] analyzed the crystal structure of SPase from *B. adolescentis* (BiSP), finding that SPase is a homodimer consisting of four domains (A, B, B’, and C) and active site residues Asp192 and Glu232, with the glucose anomeric carbon-binding site and the glucoside-binding site located in domain A [17]. After double mutation of L341I and Q345S in BiSP, selectivity for kojibiose synthesis increased from 35% in the wild-type variant to 95% in the mutant. This might be explained by the isoleucine in the L341I mutant increasing hydrophobic interactions, whereas the serine in the Q345S mutant promotes the formation of a hydrogen bond with the O4 of the acceptor glucose, leaving space for the formation of two hydrogen bonds between the hydroxyl group of C6 and Asp342. These additional interactions likely result in the increased selectivity related to kojibiose production [9].

Currently, BiSP is only heterologously expressed in *E. coli* [15, 18–20]; however, *E. coli* can produce endotoxin, making it unsuitable for use in the food industry. *Bacillus subtilis* does not secrete exotoxin or endotoxin and is considered a generally recognized as safe (GRAS) organism, exhibits no obvious codon preference, produces expression products that do not form inclusion bodies, and is capable of good protein secretion. In this study, *B. subtilis* was used as the host, and a recombinant *B. subtilis* strain was constructed to express intracellular BiSP. The recombinant enzyme was secreted extracellularly, and we obtained high levels of SPase expression, enabling straightforward enzyme isolation and purification. Effective kojibiose synthesis was performed using the crude enzyme solution from the supernatant of fermentation broth of the recombinant strain, providing a basis for potential industrial-scale preparation of kojibiose.

## Results

### Recombinant SPase expression in shake flasks

**Fig. 1:**
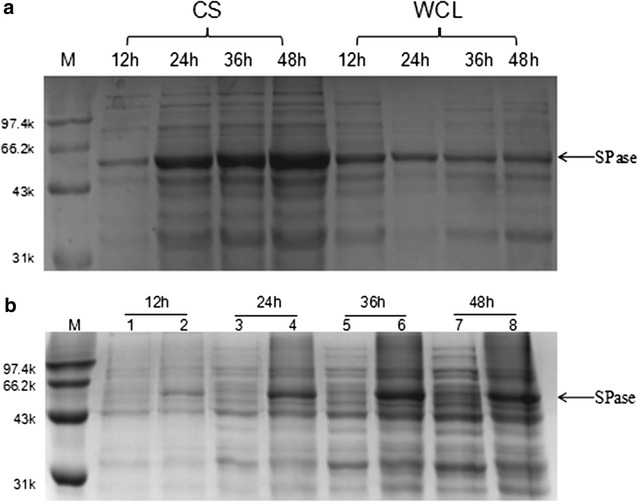
SDS-PAGE analysis of SPase expression in *B. subtilis* in flasks. **a** SPase expression in *B. subtilis* with pBSMuL3-SPase plasmid. **b** SPase expression in *B. subtilis* with pBSMuL3-SPase plasmid or blank pBSMuL3 plasmid (Lanes 1, 3, 5 and 7 are CS samples in *B. subtilis* with blank pBSMuL3 plasmid; Lanes 2, 4, 6 and 8 are CS samples in *B. subtilis* with pBSMuL3-SPase plasmid). The band of SPase (56 kDa) is indicated with an arrow. (*M* marker *CS* culture supernatant, *WCL* whole-cell lysates)

**Fig. 2:**
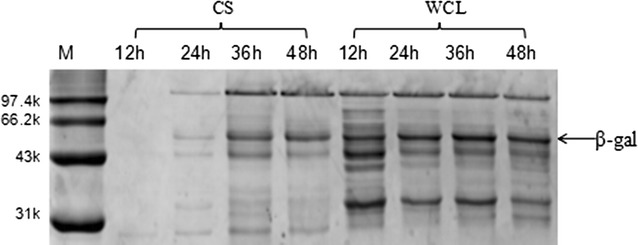
SDS-PAGE analysis of β-gal expression in the recombinant *B. subtilis* with pBSMuL3-β-gal plasmid in flask. The band of β-gal (63 kDa) is indicated with an arrow (*M* marker, *CS* culture supernatant, *WCL* whole-cell lysates)

Recombinant *B. subtilis* strains were transferred to shake flasks containing 50 mL terrific broth (TB) medium for fermentation, and enzyme activity was measured at different cultivation times. SPase is an intracellular enzyme, and the recombinant plasmids used for this study did not contain signal-peptide sequences. However, most SPases were secreted extracellularly (culture supernatant bands in Fig. 1a), with only small intracellular concentrations (whole-cell lysate bands in Fig. 1a) remaining, whereas the blank pBSMuL3 plasmid control showed no SPase band (Fig. 1b). Enzyme activities were determined both intracellularly and extracellularly (Table 1), with the activity of extracellular SPase reaching 1.58 U/mL after 48 h of cultivation and accounting for 73.6% of the total enzyme activity. The N-terminal sequencing showed that the N-terminal of extracellular SPase and intracellular SPase were identical (NKVQLITYADRLGDGTIKSMTDILRTRFDGVYDGVHILPFFTPFDGAD). We inserted β-gal (with no signal peptide) from *Sulfolobus solfataricus* into *B. subtilis* as a control, finding that no enzyme was secreted extracellularly before 24-h fermentation (Fig. 2). After 48-h fermentation, only a small amount of β-gal was observed by sodium dodecyl sulfate polyacrylamide gel electrophoresis (SDS-PAGE), indicating that SPase was more easily secreted extracellularly than β-gal. This represents the first report of extracellular SPase expression with *B. subtilis* in the absence of the signal peptide and suggested that the crude recombinant enzyme could be successfully obtained via simple treatment of the fermentation supernatant.

**Table 1:** The SPase expression from *B. subtilis* with pBSMuL3-SPase plasmid in flasks

Time(h)	12	24	36	48
OD_600_	6.5	7.4	8.5	8.3
CS SPase activity (U/mL)	0.8	1.4	1.4	1.6
WCL SPase activity (U/mL)	0.8	0.8	0.8	0.6

*CS* culture supernatant, *WCL* whole-cell lysates

Lactate dehydrogenase (LDH)-cytotoxicity assays showed that no cell damage occurred during culture (data not shown), suggesting that SPase did not exit the cells due to cell rupture. Because the enzyme was secreted in the absence of a signal peptide, this suggested that the enzyme might undergo extracellular secretion through a non-classical secretory pathway.

### Expression of SPase by recombinant *B. subtilis* in a 3-L bioreactor

**Fig. 3:**
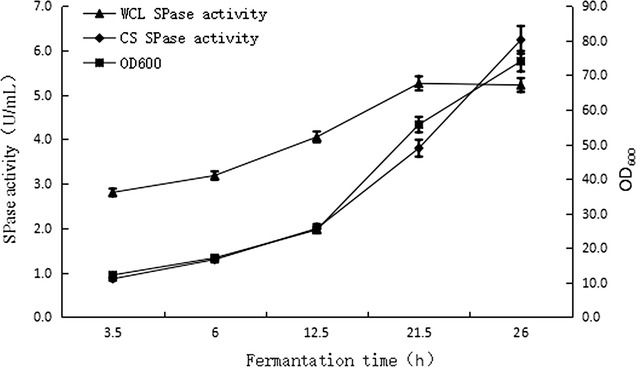
Profile of SPase activity and biomass (OD_600_) in the fermentation process of *B. subtilis* with pBSMuL3-SPase in 3-L bioreactor (*CS* culture supernatant, *WCL* whole-cell lysates)

**Fig. 4:**
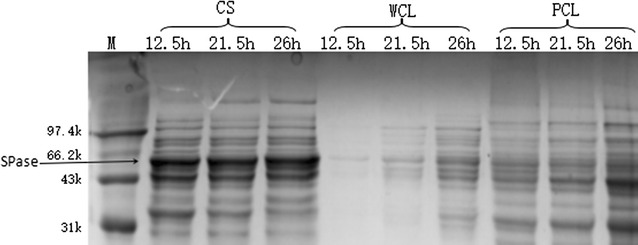
SDS-PAGE analysis of SPase expression in the fermentation process of *B. subtilis* with pBSMuL3-SPase in 3-L bioreactor. The band of SPase (56 kDa) is indicated with an arrow (*M* marker, *CS* culture supernatant, *WCL* whole-cell lysates, *PCL* precipitation of cell lysates)

As shown in Fig. 3, the highest SPase activity in the fermentation supernatant reached 5.3 U/mL after 21.5 h of cultivation in the 3-L bioreactor containing 1.2 L of optimized medium, whereas intracellular SPase activity was 3.801 U/mL, with the extracellular enzyme activity accounting for 58% of the total activity. The crude SPase content in the fermentation supernatant was 7.5 g/L, which was 3.3-fold higher than that observed in the shake flasks, with intracellular enzyme activity increasing during fermentation. The electropherogram (Fig. 4) showed that the protein bands for extracellular SPase were significantly thicker than those of intracellular SPase, indicating high secretion efficiency on the part of SPase during bioreactor fermentation. The enzymes remained inside the cell likely due to the lack of sufficient time necessary for extracellular transport. Additionally, the concentration of recombinant bacterial strains increased during the fermentation process, although enzyme activity did not change simultaneous with bacterial concentration, resulting in the highest enzyme activity being measured after 21.5 h of cultivation.

### Optimization of reaction conditions for kojibiose synthesis

**Fig. 5:**
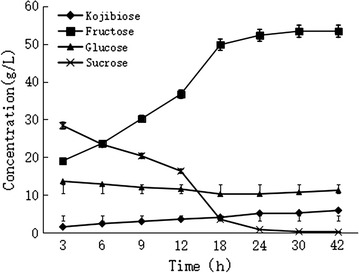
Effects of reaction time on the kojibiose production

**Fig. 6:**
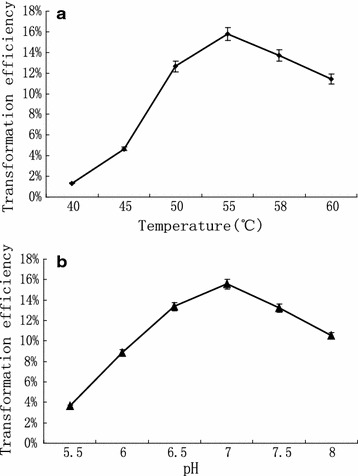
Effects of temperature **a** and pH **b** on the kojibiose production

We first investigated the effect of reaction time, with conversion rates at different reaction times determined by HPLC. The results showed that kojibiose synthesis reached equilibrium after 30 h, and that kojibiose content did not significantly increase along with prolonged reaction times (Fig. 5). Under similar reaction conditions, the reaction temperature and pH were optimized, respectively, with samples collected after 30 h. The optimized reaction temperature resulting in the highest conversion rate was 55 °C (Fig. 6a), and pH-optimization results showed that the highest conversion rate was achieved at pH 7.0 (Fig. 6b). Conversion rates were reduced under other pH conditions, likely due to the inhibition of enzyme activity; therefore, pH 7.0 was selected as the optimal reaction pH.

**Fig. 7:**
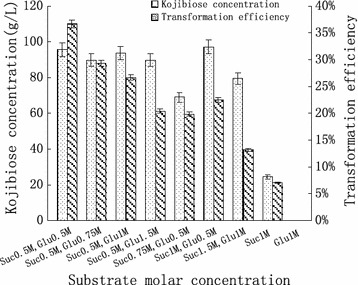
Effects of substrate concentration on kojibiose concentration and transformation efficiency

The conversion rate was evaluated by varying the molar ratio of sucrose to glucose under optimal temperature and pH conditions and at a reaction time of 30 h. The results showed that the highest conversion rate reached 38.64% when the molar ratio of sucrose to glucose was 1:1, resulting in kojibiose content in the conversion product of 95.7 g/L (Fig. 7). When the amount of glucose continued to increase, the conversion rate of sucrose declined; however, when sucrose was used as the sole substrate, kojibiose was produced at very low conversion rates.

Orthogonal test reactions were initiated by the addition of crude SPase with 0.02 U/mg_all_substrates_ with a reaction time of 30 h. The results of the orthogonal test are presented in Table 2; they revealed that the effect of three factors on transformation efficiency was in the order of A (the molar ratio of sucrose to glucose) > C (temperature) > B (pH) according to the R value, which exceeded that of the blank-column R value, indicating that the result was less affected by the error. According to the optimum K value, the optimal transglycosylation-reaction condition was A_2_B_2_C_1_ (molar ratio of sucrose to glucose: 0.5 M/0.5 M; pH 7.0; temperature: 50 °C). Based on the optimal conditions, three validation experiments were carried out, resulting in an average transformation rate of 40.01%, with the content of kojibiose at 104.45 g/L and selectivity for kojibiose production at 97%. These results were better than those associated with any combination of orthogonal tests, with these parameters subsequently determined as the optimal enzyme-conversion conditions.

**Table 2:** Orthogonal experiment result of transglycosylation reactions for kojibiose synthesis

No.	Factors				Transformation efficiency (%)
	A	B	C	Bank	
1	1	1	1	1	35.30
2	1	2	2	2	34.72
3	1	3	3	3	27.69
4	2	1	2	3	37.80
5	2	2	3	1	34.22
6	2	3	1	2	36.65
7	3	1	3	2	29.35
8	3	2	1	3	33.82
9	3	3	2	1	30.04
_K1_	0.977	1.025	1.058	0.996	
_K2_	1.087	1.028	1.026	1.007	
_K3_	0.932	0.944	0.913	0.993	
_k1_	0.326	0.342	0.353	0.332	
_k2_	0.362	0.343	0.342	0.336	
_k3_	0.311	0.315	0.304	0.331	
R	0.155	0.084	0.145	0.014	

## Discussion

In this study, BiSP was expressed in *B. subtilis* for the first time. The enzyme activity (5.3 U/mL) of recombinant *B. subtilis* SPase (pBSMuL3-SPase) was 3.5-fold higher than that (1.5 U/mL) observed in *E. coli* SPase after bioreactor cultivation [19]. The recombinant *B. subtilis* strain constructed in this study had the advantages of short culture time and high SPase-expression levels. Notably, the enzyme expressed intracellularly was capable of being secreted extracellularly in the absence of a signal peptide, with secretion efficiency reaching 73.6 and 58.1% in the flask and bioreactor, respectively. The expression plasmid used for recombinant *B. subtilis* was pBSMuL3, which reportedly expresses and secretes cutinase [21, 22], an α-amylase harboring a signal peptide [23]. In this study, the recombinant strain constructed with this plasmid in the absence of a signal peptide effectively secreted SPase extracellularly. Although this was a strange phenomenon, the extracellular expression and secretion of SPase was highly conducive to its subsequent separation and purification. Because *B. subtilis* is recognized as GRAS organism, the recombinant strain constructed in this study is suitable for large-scale preparation of recombinant SPase for use in the food industry.

SPase might be secreted via a non-classical secretory pathway. A recent study showed that some proteins, such as d-psicose 3-epimerase, glyceraldehyde 3-phosphate dehydrogenase, and pyruvate dehydrogenase E3 subunit, can be secreted extracellularly in the absence of signal peptides [24–26]. Other studies reported that these proteins might be secreted using non-classical secretory pathways rather than through processes related to cell-autolysis damage [27, 28] or by the Sec or Tat [29] pathways. Yang et al. [27] found that enolase contains a hydrophobic α-helix that promotes its extracellular secretion, indicating that this domain of the protein was involved in its secretion. Zhang et al. [24] showed that RDPE secretion might be related to hydrophobic amino acid residues located in the hydrophobic domain, which are also important for protein folding, and that non-classical secretory pathways were preferential to the secretion of multimers. SPase contains numerous hydrophobic residues and is a functional dimer, which is similar to the protein studied by Zhang et al. [24]. Therefore, it is possible that SPase might be secreted via a similar mechanism involving a non-classical secretory pathway, although the mechanisms related to non-classical secretory pathways remain unclear. Additionally, we observed that 58% of the recombinant SPase expressed intracellularly underwent extracellular secretion in the absence of cell-membrane damage, and that the conversion rate of the secreted enzyme was close to that of SPase expressed intracellularly in *E. coli*. Therefore, we speculated that SPase was fully folded intracellularly in *B. subtilis* and then secreted through a non-classical secretory pathway, the exact mechanism of which remains to be elucidated.

For kojibiose preparation, the fermentation supernatant harboring the recombinant *B. subtilis* strain expressing SPase was used as the enzyme solution, and high concentrations of inexpensive sucrose and glucose were used as the substrates. Using this system, we obtained a large amount of kojibiose over a short period of time, and by optimizing the reaction conditions, such as reaction temperature, pH, and substrate ratio, the resulting conversion rate of *B. subtilis* SPase was close to that of *E. coli* SPase, with kojibiose accounting for 104.45 g/L and a product specificity of 97%. These results indicated that this system is highly suitable for large-scale preparation of kojibiose.

## Conclusion

In this study, BiSP was expressed in a recombinant *B. subtilis* strain for the first time. Compared with SPase, which can only be expressed intracellularly in *E. coli*, we achieved efficient expression and secretion of BiSP in *B. subtilis* in the absence of a signal peptide. Enzyme activity in the culture supernatant reached 5.3 U/mL after bioreactor cultivation, accounting for 58.1% of the total enzyme activity, with SPase content in the fermentation supernatant reaching 7.5 g/L, which represents the highest reported expression of recombinant SPase. The conversion rate of the substrates sucrose and glucose by the recombinant enzyme was 40.01%, with kojibiose concentrations reaching 104.45 g/L and 97% kojibiose-production selectivity. The conversion rate and product specificity were close to those observed for SPase expressed in *E. coli*. Furthermore, we observed that intracellular SPase expression resulted in extracellular secretion, likely through a non-classical secretory pathway in *B. subtilis*. These results provide a foundation for the industrial production of kojibiose suitable for the food industry.

## Methods

### Bacterial strains and culture medium

BiSP was subjected to double point mutations (L341I, Q345S), and the DNA sequence of the mutant enzyme was synthesized chemically for use in this study. The recombinant *B. subtilis* strain expressing β-gal from *S. solfataricus* was obtained from our laboratory. The expression plasmid pBSMuL3, the DNA-manipulation strain *E. coli* JM109, and the host strain *B. subtilis* (CCTCC M 2016536) were obtained from our laboratory. The LDH assay kit was purchased from Beyotime (Shanghai, China). The pMD-19-T Simple vector, restriction enzymes (*Bam*HI and *Hind*III), bacterial DNA kit, plasmid miniprep kit, T4 DNA ligase, and agarose gel DNA purification kit were supplied by TAKARA (Dalian, China). Standard chemicals, including kojibiose, maltose, glucose, sucrose, and fructose, were purchased from Sinopharm Chemical Reagent Co., Ltd. (Shanghai, China). All other chemicals and reagents were of analytical grade.

*Escherichia coli* JM109 was used for DNA manipulation and grown in LB liquid medium (1% tryptone, 0.5% yeast extract, and 1% NaCl) or on LB agar plates (plates also contained 2% agar), each supplemented with 100 μg/mL ampicillin, at 37 °C and shaking at 200 rpm. *B. subtilis* strains were cultivated with shaking at 200 rpm and at 33 °C in TB medium containing 1.2% tryptone, 2.4% yeast extract, 1.64% K_2_HPO_4_·3H_2_O, 0.23% KH_2_PO_4_, 0.5% glycerol and supplemented with 30 μg/mL kanamycin.

The fermentation medium contained 1.5% yeast extract, 2.5% corn starch, 0.1% ammonium citrate, 0.2% Na_2_SO_3_, 0.268% (NH4)_2_SO_4_, 1.92% K_2_HPO_4_·3H_2_O, 0.4% NaH_2_PO_4_·2H_2_O, and 0.1% MgSO_4_·7H_2_O, with 4% (v/v) trace element solution (0.05% CaCl_2_, 0.835% FeCl_3_, 0.02% ZnSO4·7H_2_O, 0.01% MnSO_4_·7H_2_O, 1.005% EDTA·2Na, 0.016% CuSO_4_·5H_2_O, and 0.018% CoCl_2_·6H_2_O). Feeding medium contained: 55% d-glucose monohydrate, 0.79% MgSO_4_·7H_2_O, and 6.34% (NH4)_2_HPO_4_, with 4% (v/v) trace element solution.

### Construction of recombinant plasmid and transformation

The *SPase* gene was amplified by polymerase chain reaction (PCR) using the forward primer SP-DF (5′-GCGAAGCTTAAGGAGGATATTATGAAAAACAAAGTTCAGCTGAT-3′; the *Hind*III site is underlined) and the reverse primer SP-DR (5′-GCGGGATCCTTAAGCAACAACTGGAGGATTGG-3′; the *Bam*HI site is underlined). The PCR product and the expression plasmid pBSMuL3 were both digested with *Hind*III and *Bam*HI, gel-purified, and ligated to form the recombinant plasmid pBSMuL3-SPase, which was subsequently used to transform *E. coli* JM109 cells. The resulting expression vector (pBSMuL3-SPase) was verified by restriction analysis and sequencing, followed by transformation into the host strain *B. subtilis* by electroporation. To determine successful expression of the enzyme, we transformed a blank pBSMuL3 into *B. subtilis* as a control.

### Expression of recombinant *B. subtilis* in flasks

To create seed cultures, a single colony of the recombinant *B. subtilis* strain (CCTCC M 2016536) harboring pBSMuL3-SPase was inoculated into 10 mL LB medium supplemented with 30 μg/mL kanamycin, cultured at 37 °C for 8 h and shaken at 200 rpm. An aliquot of the seed culture (5%, v/v) was then inoculated into 50 mL TB medium supplemented with 30 μg/mL kanamycin and grown for 2 h at 37 °C with shaking at 200 rpm. The temperature was then lowered to 33 °C and incubated for another 48 h, with enzyme activity measured every 12 h. As a control, recombinant *B. subtilis* (pBSMuL3-β-gal) was cultured under the same conditions, and β-gal secretion was measured.

To analyze SPase activity, 1 mL of culture was centrifuged at 12,000*g* for 10 min at 4 °C, and the supernatant was used as the extracellular fraction. Residual whole-cell pellets were resuspended in 50 mM phosphate buffer supplemented with 20 mg/mL lysozyme, and an aliquot of the cell suspension was incubated at 37 °C for 30 min. The pellets were further lysed by sonication on ice for 10 min with a 2 s interval. Lysates were centrifuged at 12,000*g* for 5 min at 4 °C, yielding a pellet and soluble fractions. Expression of the recombinant enzyme was measured by SDS-PAGE analysis under denaturing conditions.

### Enzyme activity assay

SPase activity was determined at 55 °C, and measured according to a previously described method [19]. One unit of SPase activity was defined as the amount of enzyme causing release of 1 μmol α-glucose-1-phosphate per min under the described assay conditions.

### N-terminal sequence analysis

The intracellular and extracellular sucrose phosphorylase proteins were analyzed by mass spectrometry analysis. The mass spectrometry analysis was performed by Beijing Bio-Tech Pack Technology Company Ltd. (Beijing, China).

### Cell-membrane permeability assay

This experiment was conducted using an LDH cytotoxicity detection kit (Beyotime, Shanghai, China) according to manufacturer instructions.

### SPase production in a 3-L bioreactor

Fed-batch cultivation was performed at 37 °C for 24 h in a 3-L bioreactor (Infors HT, Bottmingen, Switzerland) [30] containing 1.2 L of optimized medium. Seed culture (100 mL LB medium containing 30 μg/mL kanamycin) was cultivated in 500-mL shake flasks and grown at 37 °C for 8 h with shaking at 200 rpm before inoculation into the bioreactor.

Dissolved oxygen (DO) and temperature were controlled at 30% and 33 °C, respectively, and phosphoric acid or ammonia solution was added, as needed, to maintain the pH at 7.0. DO, pH, temperature, and flow rates (200–700 rpm) were regulated automatically by the Infors fermentation device (Infors HT). Upon reduction of the carbon source and increase in DO in the primary medium, the feed medium was added to maintain the *B. subtilis* growth rate, thereby initiating the exponential-feeding phase of fed-batch cultivation. After fermentation the quantity of SPase was determined by scanning the area of each band on reduced SDS-PAGE gels, and then calculating with Image-Master TotalLab software (Amersham Biosciences) using purified SPase as a reference.

### Optimization of transglycosylation reactions for kojibiose synthesis

The crude SPase obtained by centrifugation of SPase-fermentation broth from the 3-L bioreactor was used for kojibiose synthesis. To determine the equilibrium time, the kojibiose-synthesis reaction was performed using 0.1 M glucose and 0.2 M sucrose as substrates and at 55 °C in 50 mM MOPS buffer (pH 6.7). Reactions with a total volume of 10 mL were initiated by the addition of 0.02 U/mg_all_substrates_ crude SPase solution. At different intervals, samples were taken and analyzed by HPLC. The reaction was terminated by boiling the mixture for 10 min, followed by centrifugation at 12,000 *g* for 20 min.

The effect of pH on kojibiose synthesis was evaluated in the pH range 5.5–8.0 at 55 °C, and the effect of reaction temperatures on kojibiose synthesis was evaluated at 40, 45, 50, 55, 58 and 60 °C under the optimal pH conditions. These reactions were performed in the presence of 0.1 M glucose and 0.2 M sucrose in 50 mM MOPS buffer for 30 h.

To determine the optimal substrate ratio, sucrose and glucose were varied from 0.5 to 1.5 M and at sucrose-to-glucose ratios of 0.5 M:0.5 M, 0.5 M:0.75 M, 0.5 M:1 M, 0.75 M:0.5 M, 1 M:0.5 M, 1.5 M:1 M, 1 M:0 M, and 0 M:1 M.

According to the results of single-factor tests, the factor level of the orthogonal test was determined, and the optimal transglycosylation-reaction conditions were determined. We applied the sucrose:glucose molar ratio (A), pH (B), and temperature (C) as study factors, with each factor having three levels, and designed the L9(3)^3^ orthogonal experiment (Table 3).

**Table 3:** The factors and levels of transglycosylation-reaction conditions in orthogonal design

Level	Factors		
	A (M/M)	B	C (°C)
1	0.5/0.75	6.5	50
2	0.5/0.5	7	55
3	0.75/0.5	7.5	60

### HPLC analysis of transglycosylation products

HPLC was used to analyze the concentrations of glucose, fructose, sucrose, maltose, and kojibiose. We used an Agilent 1200 HPLC system (Agilent Technologies, Palo Alto, CA, USA) connected to a differential refractive-index detector. A Hypersil APS2 column (250 × 4.6 mm, 5 μm; Thermo Fisher Scientific, Waltham, MA, USA) was installed in a column oven at 35 °C and was used a mixture of acetonitrile:water (75:25, v/v) as the mobile phase and at a flow rate of 0.8 mL/min. All samples were filtered through a cellulose acetate membrane (0.22 um). The conversion yield was defined as w_product_/w_all_substrates。._

## Abbreviations

SPase: sucrose phosphorylase
GRAS: generally recognized as safe
MOPS: 3-morpholinopropanesulfonic acid
LDH: lactate dehydrogenase
LB: Luria–Bertani
TB: terrific broth
SDS-PAGE: sodium dodecyl sulfate polyacrylamide gel electrophoresis
HPLC: high-performance liquid chromatography
β-gal: β-galactosidase
PCR: polymerase chain reaction
DO: dissolved oxygen
